# Power Laws Describe Bacterial Viscoelasticity

**DOI:** 10.1021/acs.langmuir.2c02172

**Published:** 2022-12-09

**Authors:** Andreas Weber, Daniel Tyrakowski, José L. Toca-Herrera

**Affiliations:** Institute of Biophysics, Department of Nanobiotechnology, University of Natural Resources and Life Sciences Vienna − BOKU, 1180Wien, Austria

## Abstract

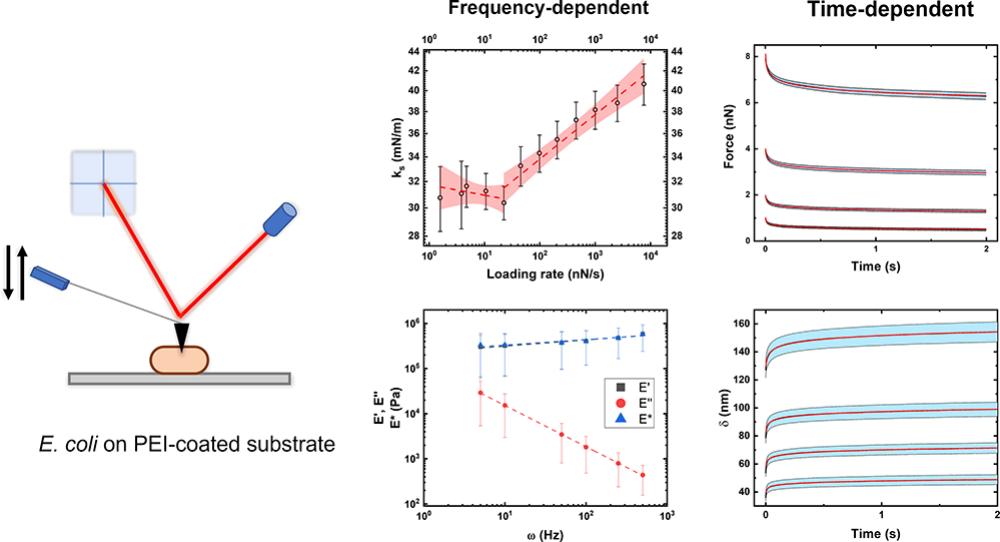

Bacterial cells survive in a wide range of different environments and actively tune their mechanical properties for purposes of growth, movement, division, and nutrition. In Gram-negative bacteria, the cell envelope with its outer membrane and peptidoglycan are the main determinants of mechanical properties and are common targets for the use of antibiotics. The study of bacterial mechanical properties has shown promise in elucidating a structure–function relationship in bacteria, connecting, shape, mechanics, and biochemistry. In this work, we study frequency and time-dependent viscoelastic properties of *E. coli* cells by atomic force microscopy (AFM). We perform force cycles, oscillatory microrheology, stress relaxation, and creep experiments, and use power law rheology models to fit the experimental results. All data sets could be fitted with the models and provided power law exponents of 0.01 to 0.1 while showing moduli in the range of a few MPa. We provide evidence for the interchangeability of the properties derived from these four different measurement approaches.

## Introduction

Bacteria are ubiquitous single cellular organisms that occur at a large diversity of ecosystems with different environments such as temperature, pH, pressure, and shear forces.^1−3^ Bacteria tightly control their shape and size to perform biological functions like growth, division, motility, and nutrient uptake.^4−8^ The main structure controlling shape and size is the bacterial cell envelope.^5^ In Gram-negative bacteria, it is made up of three layers: The cytoplasmic membrane (or inner membrane), the peptidoglycan cell wall and the outer membrane. The inner membrane is a phospholipid bilayer that encompasses membrane proteins with functions in energy production, protein secretion and transport.^9^ The peptidoglycan layer (also called cell wall) can be thought of as large polymer made up of repeating disaccharide units that are cross-linked via pentapeptides.^10^ The outer membrane is an asymmetric lipid bilayer, with the outer leaflet being made up of glycolipids (mostly lipopolysaccharide), and the inner made up of phospholipids.^11^ These three structures have distinct architectures and are interlinked with each other either covalently or noncovalently. Besides the functions named above, the layers of the cell envelope play distinct roles in resistance to substances that are toxic to the bacteria, such as antibiotics.

The envelope is the interface between the inside of the bacterial cell and its environment and is often described as a rigid body.^10^ It is the main component responsible for keeping up the positive turgor pressure between inside and outside of the cell, while withstanding osmotic pressure of a few atm.^12^ Mechanical properties of bacterial cells (and their envelope) have recently become a focal point of biophysical studies as they are integral for survival of the cells and their growth.^2,6,13^ Diverse antibiotics attack either directly the cell envelope or the biochemical machinery that constructs the cell envelope, and therefore bacterial mechanics are thought to be essential parameters to consider. In addition, for cell division and growth, bacteria must actively invest energy to overcome the pressure of the surrounding environment while keeping up their shape.^8^

Methods to study bacterial mechanics include optical microscopy combined with osmotic shocks, microfluidic devices, magnetic and optical tweezers, or growth assays in gels with defined mechanics.^13^ Probably the most powerful technique employed for elucidating bacterial mechanics is atomic force microscopy, as it enables three-dimensional topographic imaging with nanometric resolution and mechanical measurements at the same time.^14^ AFM has been applied in microbiological surface properties studies for investigating phage infection of bacteria,^15^ connecting surface and interfacial properties with antibiotic resistance^16^ and application of DLVO theory for describing interactions between bacteria and surfaces.^17,18^ Recently, AFM has been used to study turgor pressure, elastic, and viscoelastic properties of both Gram-positive and Gram-negative cells.^3,19−22^ Time-dependent measurements together with the use of simple spring–dashpot combination models such as the standard linear solid were used to determine viscoelastic properties of bacteria.

In this work, we study the frequency and time dependent mechanical properties of Gram-negative bacteria using four different AFM force spectroscopy methods. We have used force cycles at different frequencies and oscillatory microrheology, as well as stress relaxation and creep measurements, at different applied forces. We then apply simple power law rheological models to investigate the interchangeability of the measurements and models. We show that power laws are able to describe bacterial viscoelastic properties while retaining a small number of model parameters. In comparison to eukaryotic cells, bacteria are 3 orders of magnitude stiffer and behave more like an elastic solid.

## Experimental Section

### Cell Preparation

*E. coli* BL21 DE3 strains were grown overnight with constant shaking at 37 °C in lysogeny broth (LB). They were then diluted 1:250 and incubated for another 2 h in fresh medium at 37 °C to prepare cells in the exponential growth phase. For sample preparation, 1 mL of the bacterial suspension was centrifuged for 5 min at 5000 rpm, washed in PBS thrice, and the pellet was resuspended in 0.5 mL of PBS.

### Glass Slide Preparation

Circular glass coverslips (24 mm diameter) were washed with Milli-Q water and 70% ethanol and dried with nitrogen. They were then plasma-cleaned for 1 min and rinsed with PBS. They were functionalized with a 0.2% solution of polyethylenimine overnight at room temperature, then washed three times with PBS, and stored until use at 4 °C. A drop of bacterial suspension was added and left to adhere for 30 min, followed by washing steps with PBS.

### AFM Measurements

For measurements, a JPK Nanowizard III (Bruker) placed on an inverted optical microscope (Zeiss AxiObserver Z1) was used. All measurements were performed in a liquid measurement chamber with PBS at 20 °C. Triangular MSCT-E cantilevers (Bruker) with a nominal stiffness of 0.1 N/m, a resonance frequency of 38 kHz in air, and a pyramidal tip of 10 nm radius were used. Cantilevers were cleaned in ethanol, dried, and cleaned with UV/O for 30 min. Prior to measurements, calibration was done using the thermal tune method.^23^ The measurement region was defined using the microscope, then a 20 μm × 20 μm AFM low resolution image was done using the quantitative imaging mode (100 μm/s approach/retract rate, 1 μm curve length, 0.3 nN force set point, 0.1 MHz data acquisition rate, 100 × 100 pixels) to localize bacteria.

**Figure 1:**
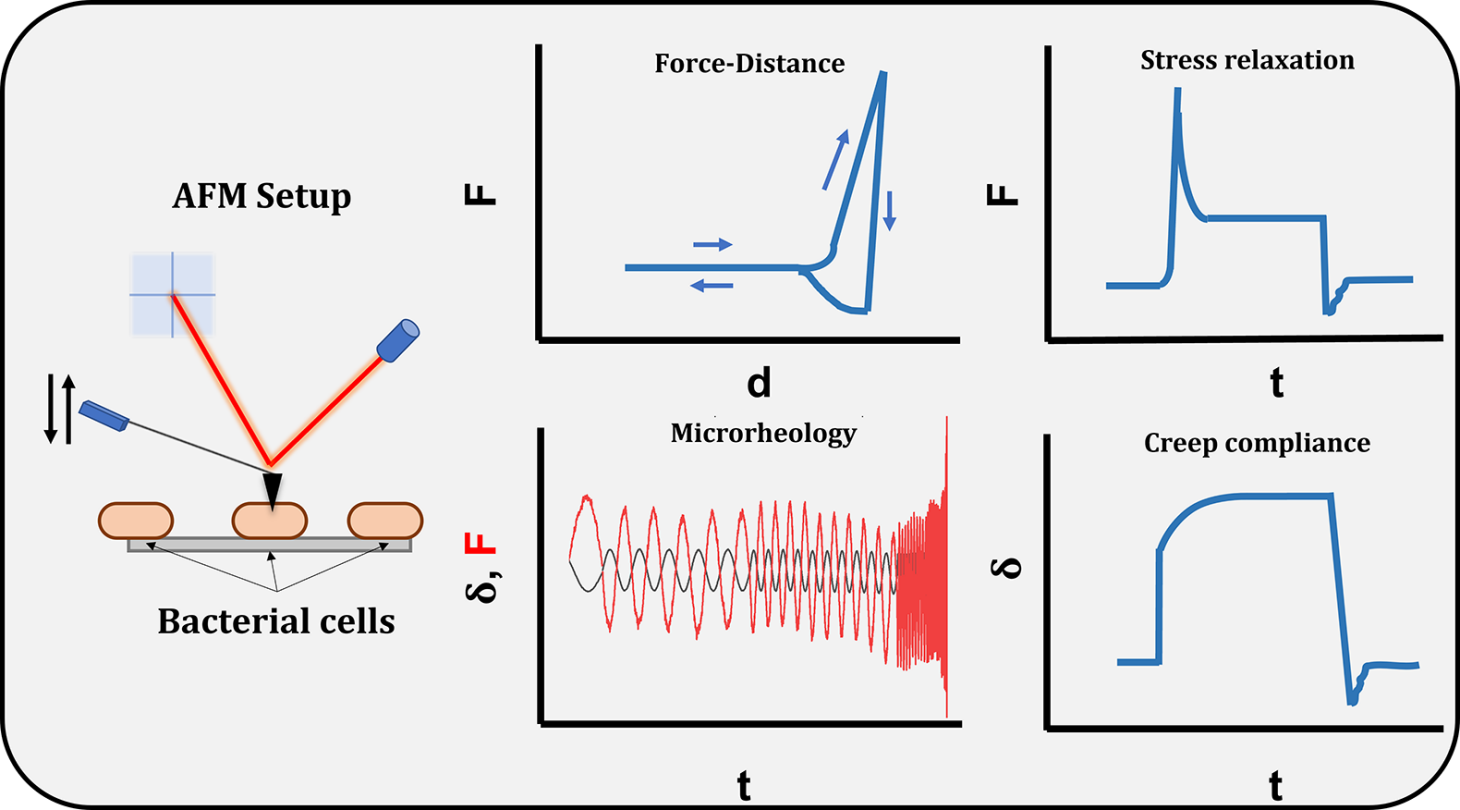
Measurement summary. The left side depicts the AFM setup used. The four insets on the right side show the types of measurements performed: force–distance (also called force-cycle), oscillatory microrheology, stress relaxation (constant deformation), and creep compliance (constant force).

Four types of mechanical measurements were performed: force–distance cycles, microrheological oscillations, stress relaxation, and creep. For force–distance measurements, a curve length of 1 μm, an approach rate of 10 μm/s and a force of 1 nN was used. Per cell, a map of 200 nm× 200 nm was measured at the planar center of the cell with a resolution of 4 × 4 pixels. The approach rate was varied from 0.0625 up to 128 μm/s to test frequency dependence. To evaluate the frequency dependent complex modulus of the *E. coli* cells, oscillatory microrheology measurements were performed. After QI imaging, a central region of the bacterial cells was probed by a 2 × 2 force map. Cells were indented to force set point of 1 nN, then a stress relaxation segment of 5 s was applied. Then the sample was probed by application of a sinusoidal excitation signal with an amplitude of 5 nm and frequencies ranging from 0.5 to 500 Hz. The phase shift Δφ between the deflection signal and the excitation signal was determined. For stress relaxation measurements, the indentation was held constant for 2 s at starting forces of 1, 2, 4, and 8 nN, with a map of 2 × 2 pixels. For creep measurements, the force was held constant for 2 s at starting forces of 1, 2, 4, and 8 nN with a map of 2 × 2 pixels. At least 20 bacteria were investigated using three independent samples. Figure 1 shows the different measurement routines that were performed.

### Data Processing

Curves were extracted using the JPKSPM software, and further steps were done in the R package afmToolkit.^24,25^ Basic processing included definition of contact and detachment point, correction of baselines as well as the cantilever deflection. The indentation segments of the curves were fitted using the Sneddon extension of Hertzian mechanics for a pyramidal indenter as
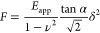
1where *F* is the applied force (N), *E*_app_ is the apparent Young’s modulus (Pa), ν is the Poisson’s ratio (set to 0.5 for an incompressible material), α is the face angle of the pyramid, and δ is the deformation of the bacterial cell (we only evaluated the first 10 nm of the curves). Like the analysis performed by Vadillo-Rodriguez,^21^ we evaluated the stiffness *k*_s_ of the bacterial cells over the whole force-distance-curve as
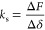
2In viscoelastic materials, the measured Young’s modulus depends on the frequency of deformation. Here, we use a simple power law to describe the approach rate dependent modulus as
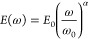
3Here *E*_0_ is the modulus at rest, ω is the measurement frequency, ω_0_ is an arbitrary frequency set to 1 s^–1^, and α is a weak power law exponent.^26,27^ In a second approach, oscillatory microrheology measurements were fitted. As the bacterial cell behaves like a viscoelastic material, both the phase shift and the deflection amplitude change with the frequency of the oscillation. The complex Young’s modulus E*(ω) consists of the storage modulus *E*′(ω) as the real part and the loss modulus *E*′′(ω) as the imaginary term as
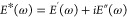
4where ω is the angular frequency. For the pyramidal indenter geometry for small amplitudes the complex Young’s modulus is
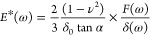
5where δ_0_ is the indentation at the beginning. This expression can be rewritten for small amplitudes as
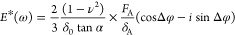
6Here *F*_A_ and δ_A_ are the force and deformation amplitudes and Δφ is the phase shift between both signals. The ratio of loss to storage modulus is called the loss tangent
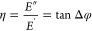
7

The loss tangent indicates whether the material shows solid (<1) or liquid-like (>1) behavior. Finally, the force response of the cantilever needs to be corrected for the hydrodynamic drag acting on the cantilever as
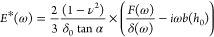
8with *b*(*h*_0_) being the drag coefficient at the surface that was extrapolated from oscillations in the viscous medium away from the surface.^28^

### Evaluation of Time-Dependent Measurements

For viscoelastic bodies, a further approach is to use a time-dependent function such as the relaxation modulus *E*(*t*) when applying a constant deformation and monitoring the decay in stress, or the creep compliance *J*(*t*) when performing creep experiments.^29^ These two properties are connected in the Laplace domain^30^ as . Assuming a linear viscoelastic body, instantaneous deformation and making use of the correspondence principle,^31−34^ the analytical solution for the stress relaxation in AFM experiments can be found as
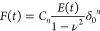
9Here,  and *n* = 2 depend on the geometry of the indenter and δ_0_ is the constant deformation of the sample. Similarly, the creep response can be written as
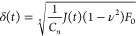
10where *F*_0_ is the constant force. Note that, for creep measurements, the indentation depth increases during the measurement, which can induce errors in analysis. In principle, these two measurements should agree with each other. Finally, analytical solutions for both the relaxation modulus and the creep compliance need to be found. Up to know, for bacterial mechanics, an arrangement of spring and dashpot models was used (e.g., as standard linear solid with a three-element model or as Burgers model).^22,35^ These models must be used in different representations (either Maxwell or Kelvin), depending on the type of measurement, which makes comparison not straightforward. We therefore decided to use simple power law models for defining both functions, making use of the continuous relaxation spectrum. For stress relaxation measurements, this leads to
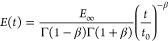
11and for the creep compliance
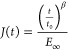
12Here Γ denotes the gamma function, *E*_∞_ is the equilibrium modulus, and β is a power law exponent that is between 0 and 1. When it is 0, the material behaves as a pure elastic solid, while when it is 1, it behaves as a pure Newtonian fluid.

## Results and Discussion

### Elastic Properties Show Biphasic Frequency Dependence

**Figure 2:**
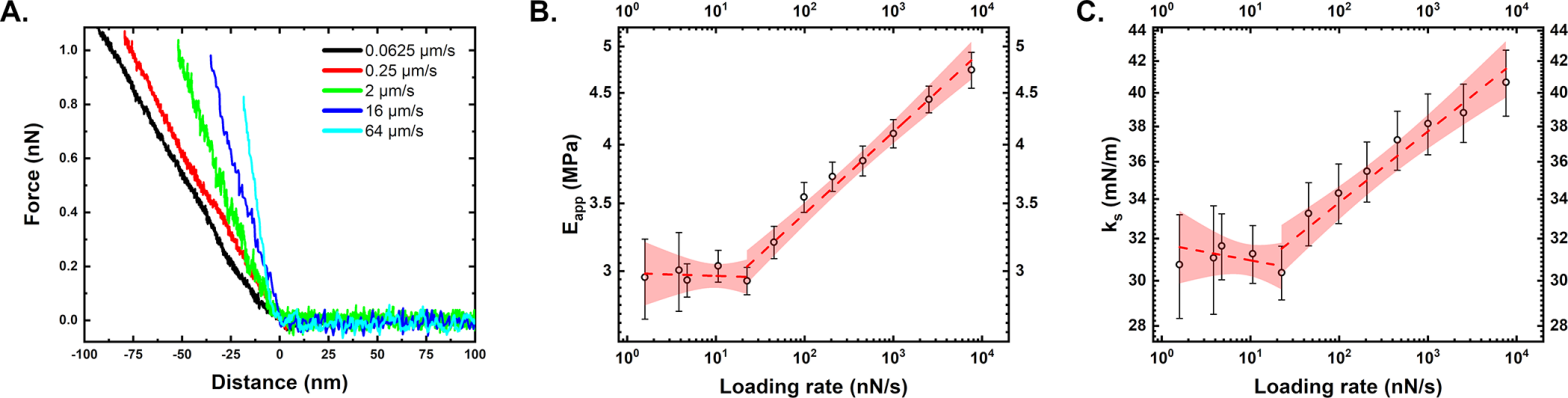
Elastic properties of *E. coli* at different frequencies. (A) Representative force–distance curves at increasing frequencies. (B) Young’s modulus dependence on frequency. (C) Stiffness dependence on frequency. In B and C, the initial red line indicates a linear fitting in the low frequency range. The dashed line indicates the power law fitting of the region from 20 to 7600 nN/s (1–128 μm/s). The red area indicates a confidence interval of 0.05.

In a first step, we performed force cycle measurements on immobilized bacteria with loading rates ranging from 0.0625 up to 128 μm/s (being 1.6 to 7600 nN/s in the present setup). These correspond to effective frequencies (expressed as , where *t*_F_ is the time needed to reach the force set point) of 0.71 up to 7470 Hz. Figure 2 shows the results of this analysis. In Figure 2A, a set of force–distance curves performed at different loading rates can be seen. Note the increase in slope with an increase in loading rates. In addition, due to hydrodynamic drag, the maximum force reached in the curves decreases with frequency. Then, elastic theory was used to determine either the Young’s modulus for the first 10 nm of the indentation segment or the stiffness from the whole indentation (Figure 2B,C). The Young’s modulus was in the range of a few MPa, which agrees well with the values published in the literature for measurements performed at similar frequencies with sharp pyramidal tips. In between approach rates of 1.6–20 nN/s (0.0625 up to 1 μm/s), the modulus behaved roughly constant, being close to 3 MPa. For the approach rate range of 20–7600 nN/s (1 up to 128 μm/s), an increase in Young’s modulus to values of up to 5 MPa can be seen. This scaling was fitted using a simple power law, as introduced in eq 3. A power law exponent of 0.081 ± 0.005 and a modulus at rest of 2.36 ± 0.07 MPa were determined from the fittings (*R*^2^ of 0.98).

Figure 2C shows a similar analysis performed with the stiffness values derived from the whole indentation segments. Stiffnesses at the low frequency plateau had values of around 0.031 N/m. Again, in the range of 20–7600 nN/s (1–128 μm/s), a nonlinear scaling could be seen that was described by a power law with an exponent of 0.047 ± 0.004 (*R*^2^ of 0.93). For the highest rate of 7600 nN/s, the stiffness was close to 0.041 N/m. These values agree well with data provided in the literature.^22,36^

The scaling of the measured mechanical properties of biological materials with frequency is a well-established observation for systems such as cells and bacteria, as they are viscoelastic.^22,37,38^ Often, this is described by the complex modulus that has a real (storage modulus, elastic property) and an imaginary (loss modulus, dissipative, viscous property) component, with both quantities depending on frequency. Our data provides evidence that, at low frequencies, the measured mechanical properties stay constant, while they show a scaling behavior at higher frequencies. Vadillo-Rodriguez^22^ investigated a similar system, applying a standard linear solid (SLS) model to creep curves and performing indentation measurements at effective frequencies of 0.5 up to 80 rad/s (0.35–72 nN/s, with a maximum load of 6 nN). They then compared the frequency domain solution for the SLS with stiffness values for the storage modulus and hysteresis values for the loss modulus derived from force–cycle measurements. In their evaluation, they only see a rise in the storage modulus with frequency, and no plateaus can be seen. Similarly, Dague^37^ investigated the use of different imaging modes (QI, QNM, FV), and saw a scaling of the derived moduli with frequency. The explanation for the plateau of measured elasticity at low frequencies is that the measurement time is longer than the characteristic relaxation time of the bacterial cell surface, and therefore, relaxation occurs during the measurement. We were limited in our AFM system to a maximum loading rate of 128 μm/s.

### Microrheological Oscillations Show Storage Modulus Dominates Mechanical Properties

**Figure 3:**
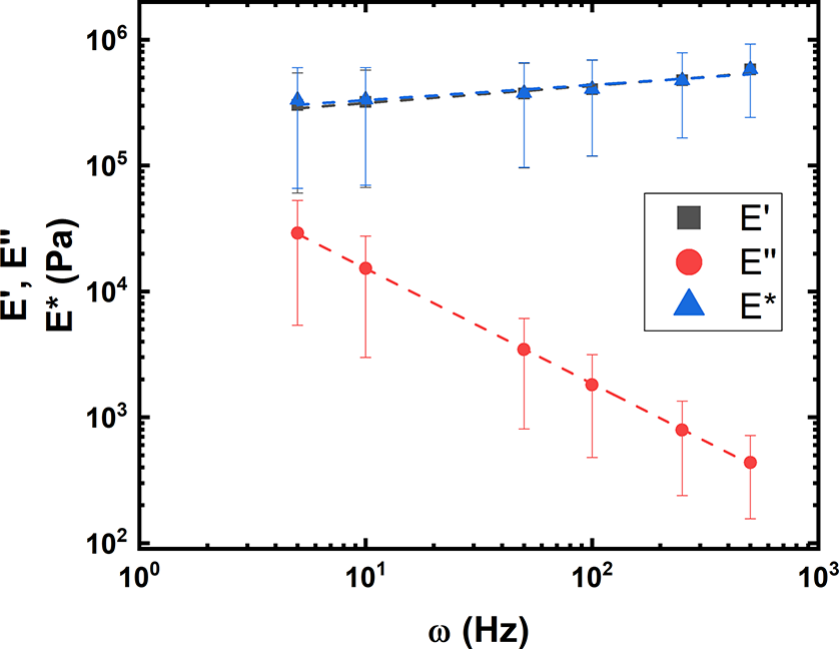
Storage (*E*′), loss (*E*′′), and complex modulus (E*) of *E. coli* cells derived from oscillatory microrheology with an applied amplitude of 5 nm and a frequency from 5 to 500 Hz. The fittings follow a power law. The error bars correspond to the standard deviation.

Oscillatory microrheology was used to study the frequency dependence of the storage, loss, and complex modulus from 5 to 500 Hz, with a deformation amplitude of 5 nm. As expected, the amplitude of force increased with applied frequency. Figure 3 shows the results of this analysis. The response was separated in the real and complex part. The former appeared constant at low frequencies and increased slightly at frequencies higher than 50 Hz, with a power law scaling of 0.138 ± 0.018 (*R*^2^ = 0.93). The loss modulus decreased with frequency following a power law with an exponent of −0.914 ± 0.005 (*R*^2^ = 0.99). At 5 Hz, the loss tangent was 0.1, decreasing significantly with frequency. Therefore, the complex modulus at high frequencies is determined by the storage modulus, which indicates that the bacterial material behaves more solid-like, underlining the above analysis.

Microrheology oscillations measurements have been used for eukaryotic cell mechanicals analysis showing power law exponents in the range of 0.15 to 0.4 and the application of a structural damping model.^39−42^ To our knowledge, the present study applies this approach for the first time to investigate bacterial mechanics. Compared to eukaryotic cell mechanics, we report striking differences: Mammalian cells show a scaling of both storage and loss modulus with frequency, possessing a crossover frequency where the viscous dissipation becomes higher than the elastic response. In the present case, the loss modulus is always lower than the storage modulus, and for higher frequency its contribution to the complex modulus tends toward zero. Furthermore, the loss modulus decreases with frequency. This indicates that in the frequency range studied here, bacterial cells behave as more like viscoelastic solids. The most often applied theory to discuss microrheological measurements of soft matter is soft glassy rheology (SGR).^43^ In the framework of this theory, at very low frequencies, there is a crossover between storage and loss modulus, after which the viscous component decreases. This behavior stems from possible slow relaxation modes at very low frequencies. Compared to mammalian cell mechanics, the frequencies probed here are beyond the glass transition temperature (which is inverse to the frequency) of the material. Additionally, at these low frequencies, the frequency dependent behavior of storage and loss modulus is similar to what one would expect from both a maxwell element, a standard linear solid as well as a generalized maxwell model, as published in an investigation of the dynamic mechanical behavior of bacterial surfaces and for measurements of eukaryotic cells.^21,44^

### Stress Relaxation Can Be Described by Power Laws at Different Loads

**Figure 4:**
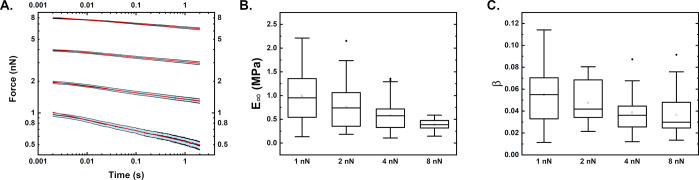
Viscoelastic properties of *E. coli* cells derived from stress relaxation experiments. (A) Average force–time curves for measurements performed at initial forces of 1, 2, 4, and 8 nN (corresponding to constant deformations of 50, 80, 140, and 220 nm) with standard error. The red line indicates the power law rheology fitting. The plot shows force vs time in logarithmic scaling. (B) Equilibrium moduli derived by using the PLR fitting. (C) Power law exponents derived from the fitting. The boxplots range from the 25th to the 75th percentile, with the mean value shown as a rectangle and the median as a line. B and C show the values derived for initial forces of 1, 2, 4, and 8 nN.

In a next step, we performed nanomechanical stress relaxation measurements on the bacteria by keeping a constant deformation at 1, 2, 4, and 8 nN for 2 s making use of the AFM feedback mechanisms. Note that these force set points correspond to initial deformations of 50, 80, 140, and 220 nm, resulting in initial stiffness values of 0.02, 0.025, 0.029, and 0.036 N/m. The curve traces showed a force decay over the measurement time that could be very well fitted by a power law rheological model with *R*^2^ values of above 0.99 (Figure 4A). From these fittings, we then evaluated the relaxation modulus (considering the geometry of indentation) and the power law exponent.

The calculated equilibrium modulus decreases with the applied force and ranges from 1 MPa at 1 nN to around 0.5 MPa at 8 nN. Together with the increase in stiffness with initial force, this observation can be explained by the bacterial cell being a multilayered structure with both the outer membrane and the peptidoglycan as rigid, stiff materials. Therefore, at lower initial forces that correspond to lower deformations, the material response is dominated by the relaxation of these layers. For higher forces, a larger volume of the cytoplasm is deformed that is assumed to behave more like a viscous fluid. The power law exponent ranges from 0.05 at initial forces of 1 nN to around 0.04 at 8 nN. There is only a slight, not significant reduction in the exponent. To our knowledge, this is the first report of stress relaxation experiments performed on bacterial cells. Still, comparing the derived properties with viscoelastic moduli of bacteria in literature, similar values have been published.^22^

### Creep Behavior Follows Power Law

**Figure 5:**
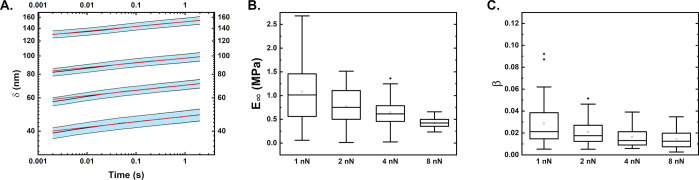
Viscoelastic properties of *E. coli* cells derived from creep measurements. (A) Average deformation–time curves for measurements performed at forces of 1, 2, 4, and 8 nN with standard error. The red line corresponds to the power law fitting. The plot shows the deformation vs the time in a logarithmic scale. (B) Equilibrium moduli derived by using the PLR fitting. (C) Power law exponent derived from the fitting. The boxplots range from the 25th to the 75th percentile, with the mean value shown as rectangle and the median as line. The values in B and C are derived from creep measurements at 1, 2, 4, and 8 nN.

Finally, we performed creep experiments at constant forces of 1, 2, 4, and 8 nN while monitoring the increase of deformation over 2 s. The deformation increased monotonically with time and the overall creep magnitude was 11, 16, 18, and 22 nm for 1, 2, 4, and 8 nN respectively. An increase in the creep response shows that the material does not behave like a linearly viscoelastic one but rather at different indentation depths, different properties are felt. Figure 5 shows this analysis. In Figure 5A, the averaged deformation-time curves can be seen together with a power law rheological fitting (*R*^2^ of above 0.99). For initial forces of 1 and 2 nN, there appears to be a slight deviation of the fitting to the data in the initial time region. These fittings were then used to determine the creep compliance and the power law rheological exponent.

The determined creep compliance decreased with applied force and ranged from 1 MPa for 1 nN to around 0.5 MPa for 8 nN. We again assume here that this effect is present due to the multilayered structure of the cells. The power law exponent ranged from 0.03 to 0.02 and appears to nonsignificantly decrease with applied force. Comparing the creep response to the stress relaxation one, the calculated moduli are similar, while the power law exponent is lower for creep experiments.

### Power Law Rheology in Biological Materials Mechanics

In this work we have used simple power law rheological models to determine the viscoelastic properties of Gram-negative bacterial cells. To our knowledge, this is the first work discussing bacterial mechanics in this light, as other researchers have mostly applied spring or spring–dashpot models to describe the frequency and time-dependence of bacterial mechanics. Power law rheological models are based on the idea of soft glassy rheology, where the exponent is a dimensionless number that indicates the degree of solidity or fluidity, respectively.^40,43,46^ An exponent of 0 corresponds to an elastic solid, while one of 1 corresponds to an ideal Newtonian fluid. In such models, the cells are thought to be made up of an ensemble of many disordered elements that exist in energetic traps. These wells are thought to be shallow, allowing for spontaneous “hopping” out. Such models have been widely applied in eukaryotic cell mechanics, as the eukaryotic cytoskeleton is thought to deform, flow and remodel being a soft glassy material close to glass transition. Other materials of this group include foams, colloidal suspensions, emulsions, and slurry. Interestingly, for eukaryotic cells, the complex modulus, stress relaxation response and creep response all follow a power law over time or frequency in the range of small deformation amplitudes. Eukaryotic cells typically show exponents ranging from 0.1 to 0.4.^34,42,47−49^ The estimated power law exponents for the bacterial cells in this study range from 0.01 up to 0.13. This is an intuitive result, as bacterial cells are thought to behave more like elastic solids than mammalian cells due to the structure and organization of their cell envelope.

## Conclusions

We have shown that the frequency and time dependence of the mechanical properties of Gram-negative bacteria can be readily described by simple power laws reducing the number of necessary fitting parameters that other models use. For force-cycles, at low frequencies a constant modulus was measured while for indentation rates from 1 to 128 μm/s, a power law scaling with the factor 0.09 for the modulus and 0.05 for the stiffness were found. Similar scaling trend was determined for oscillatory microrheological measurements as well as stress relaxation and creep data. Novel application of such models to stress relaxation and creep measurements showed similar exponents with relaxation moduli and creep compliance values of around 1 MPa. For future work, disturbing of the bacterial surface and cell envelope structure with evaluation of power law properties will be promising. Furthermore, the correspondence between bacterial mechanics and their surface properties are intertwined factors to consider for bacterial biofilm formation.
